# Supplementary material for: A massively parallel reporter assay library to screen short synthetic promoters in mammalian cells
Source: Nat Commun. 2024 Nov 28;15:10353. doi: 10.1038/s41467-024-54502-9 (PMC11604768; doi:10.1038/s41467-024-54502-9)
Supplement: Supplementary file 1 — Supplementary Information [file 41467_2024_54502_MOESM1_ESM.pdf]

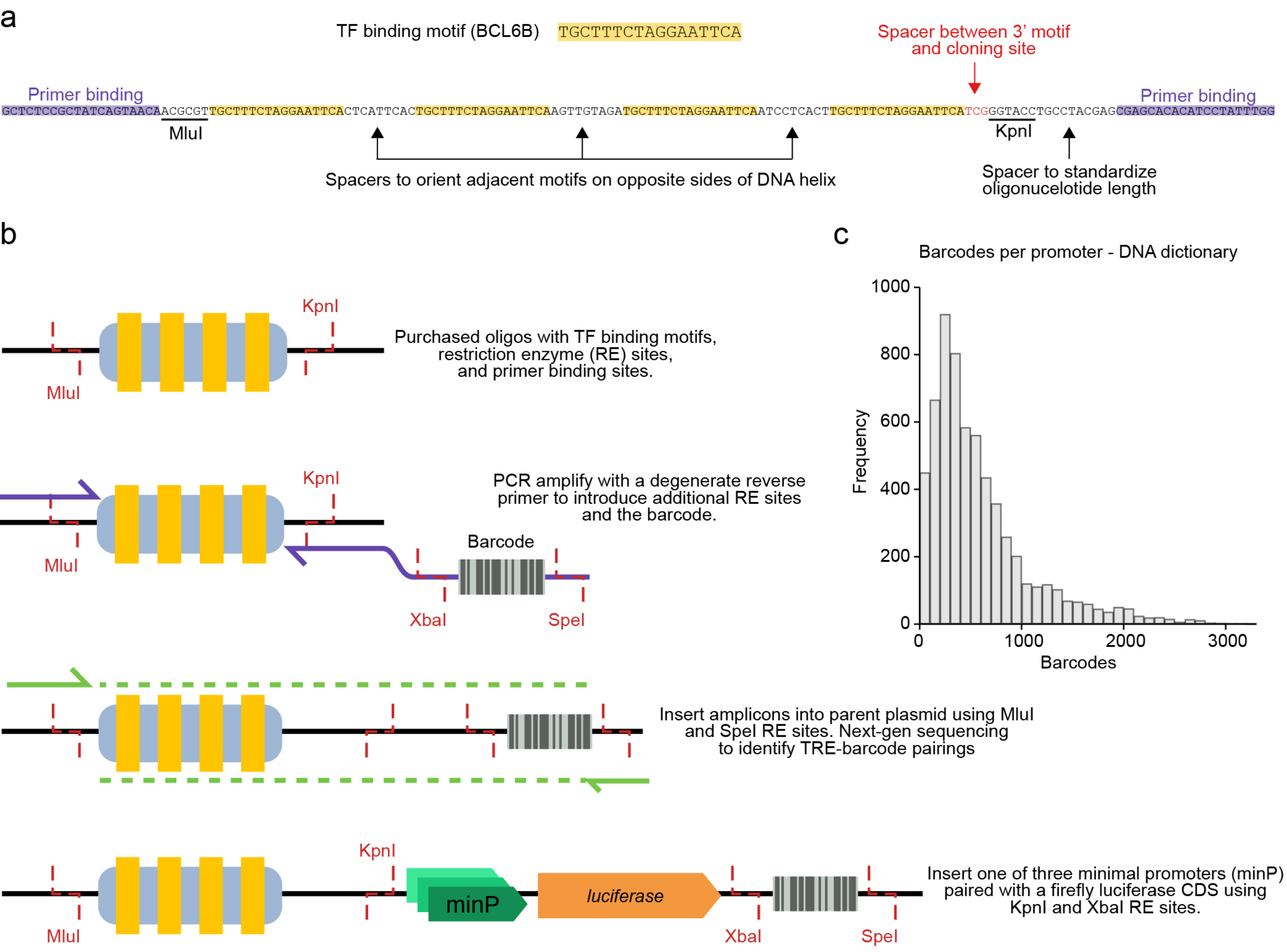

Supplementary Figure 1 - TRE unit design and promoter production. a Schematic of the TRE unit oligonucleotides used for MPRA library cloning. **b** Graphical overview of TRE-MPRA library production. **c** Histogram of the barcodes per promoter in the plasmid library barcode dictionary as determined by next-generation sequencing.

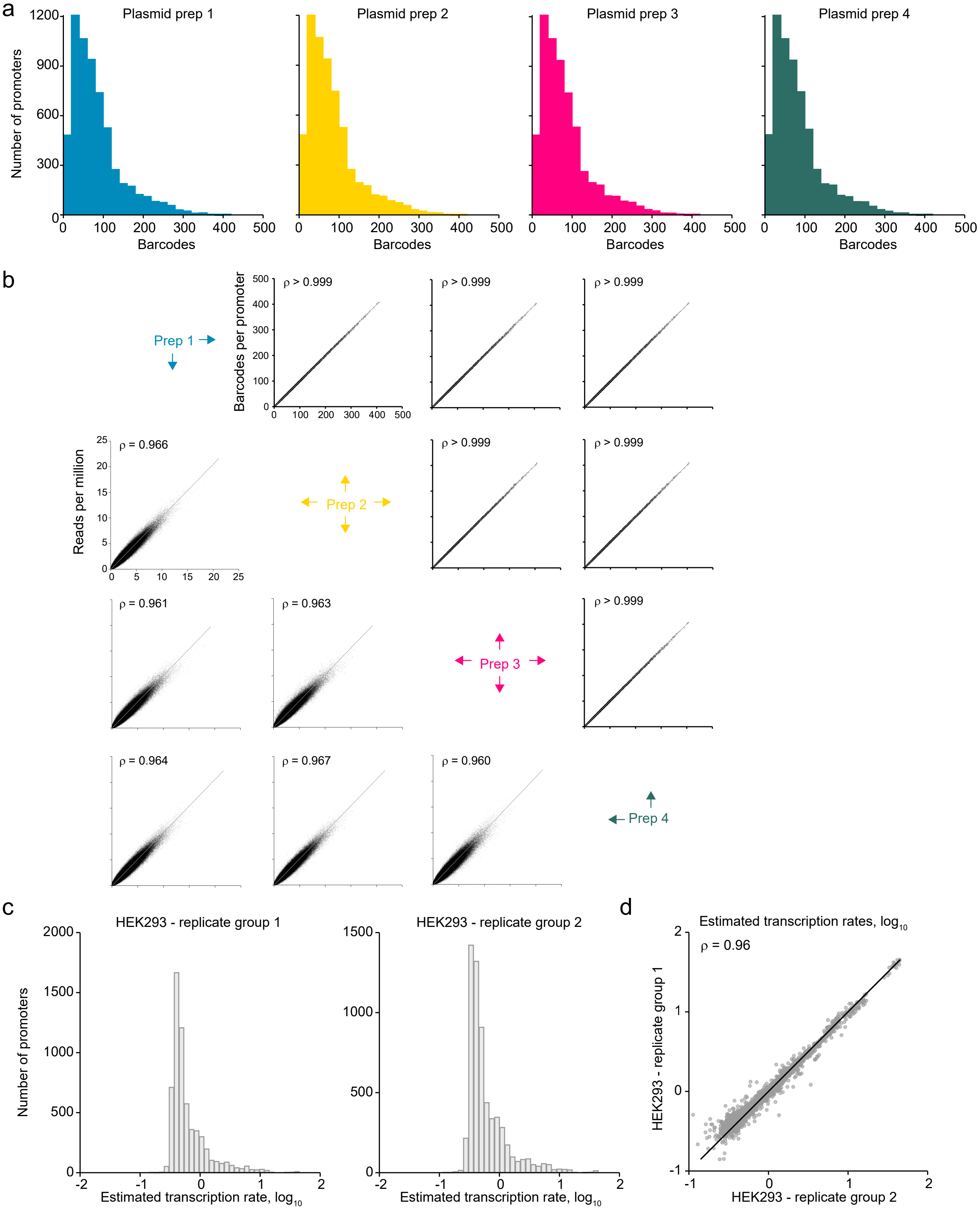

Supplementary Figure 2

Supplementary Figure 2 - Comparison of independent TRE-MPRA plasmid library preparations. a The number of barcodes per promoter from four independent TRE-MPRA plasmid libraries prepared from distinct bacterial liquid cultures. **b** Independent plasmid libraries showed highly correlated barcodes per promoter (upper right panel) and promoter reads per million (lower left panel) as determined by NGS. ρ, Spearman's correlation coefficient. **c** Histograms of the estimated transcription rates in untreated HEK293 cell replicate groups. **d** Scatterplot comparing transcription rate estimates ($\log_{10}$) between untreated HEK293 cell replicate groups. Solid black line indicates the identity line (y=x). ρ, Spearman's correlation coefficient.

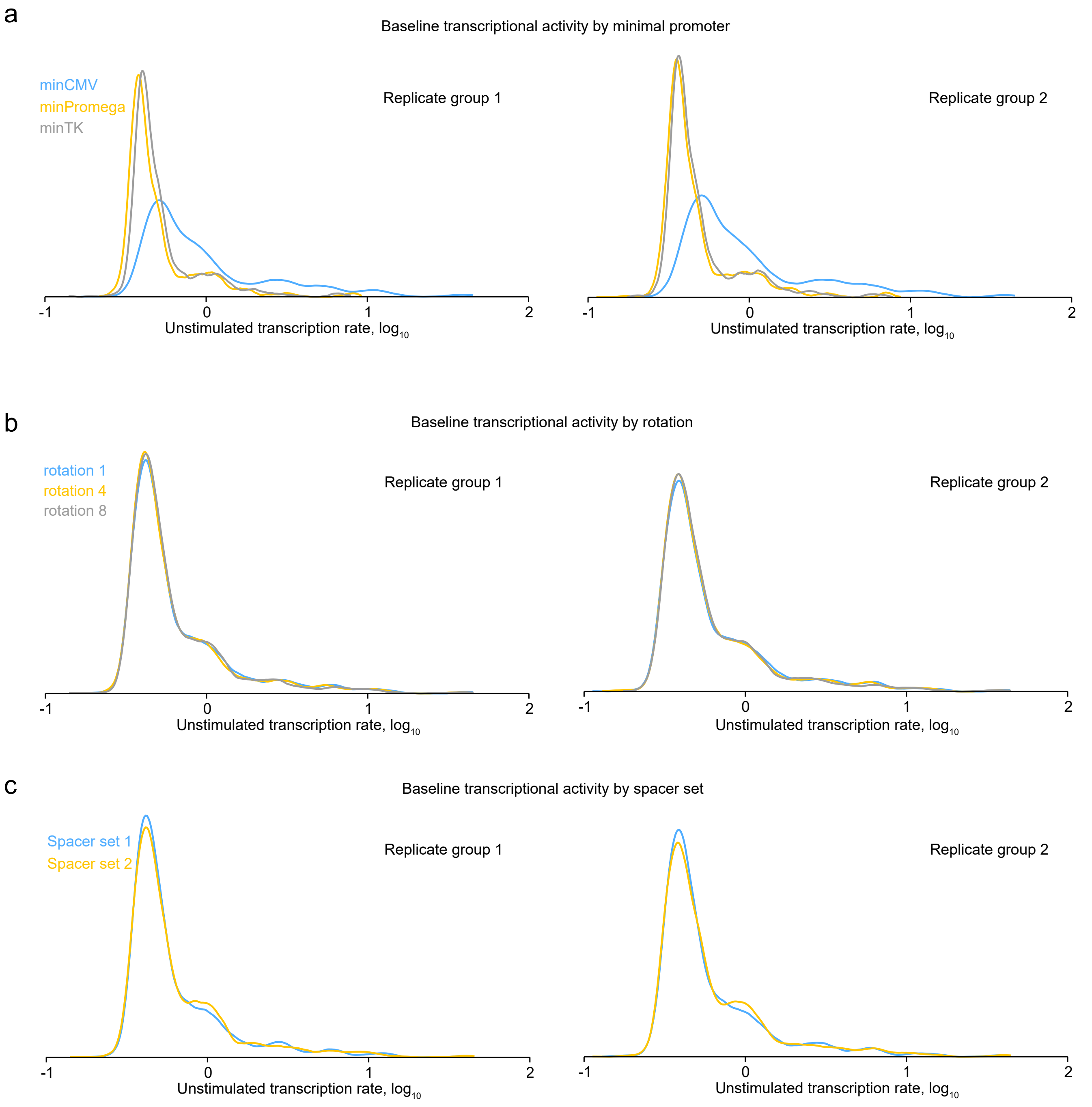

Supplementary Figure 3

Supplementary Figure 3 - Effects of promoter design on transcription rates. a Density plots of baseline transcription rate estimates for each minimal promoter across two independent sets of untreated HEK293 replicates. **b** Density plots of baseline transcription rate estimates for each nucleotide distance between TRE units and the minimal promoter across two independent sets of untreated HEK293 replicates. **c** Density plots of baseline transcription rate estimates for each of two spacer sets across two independent sets of untreated HEK293 replicates.

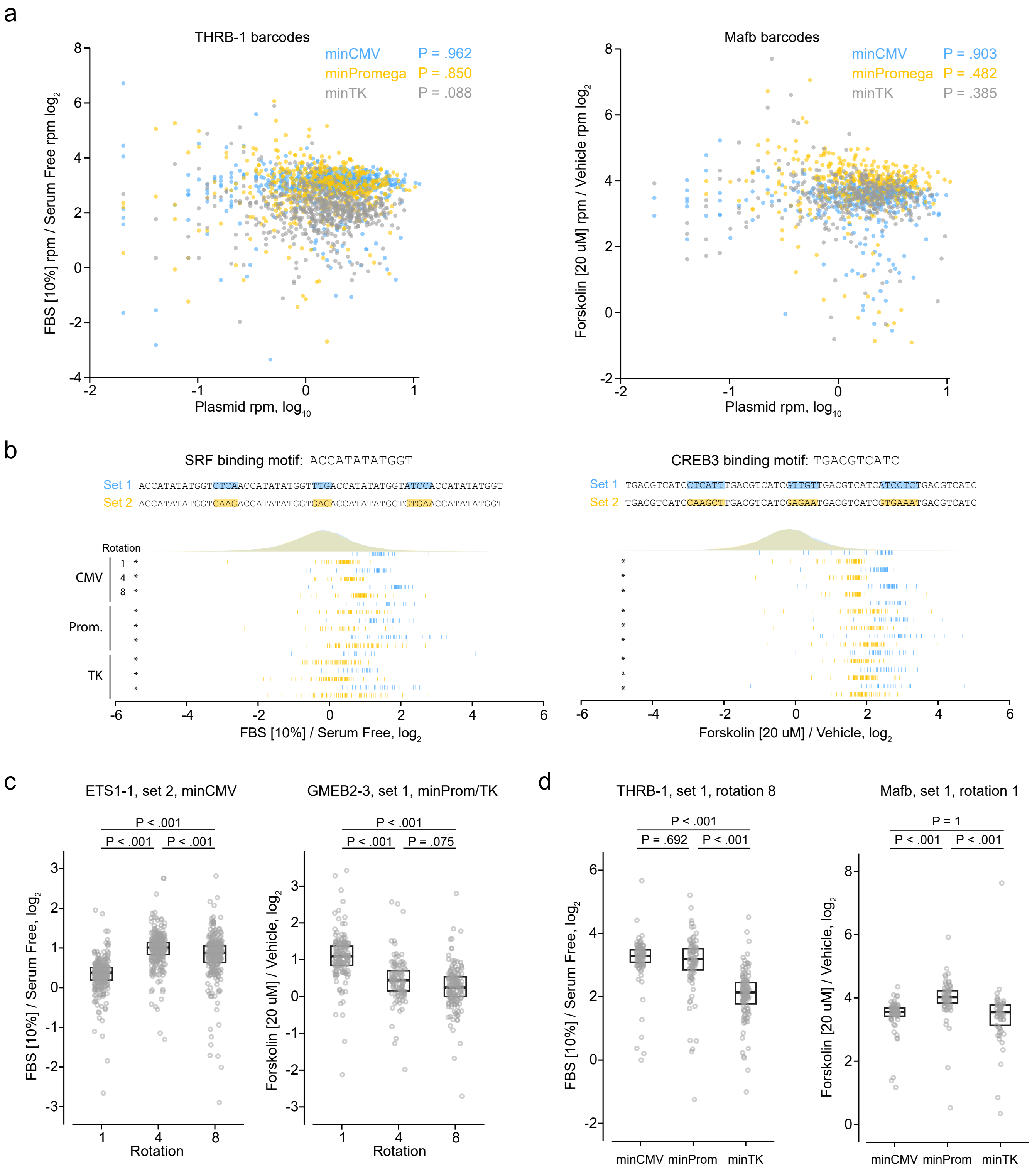

Supplementary Figure 4

Supplementary Figure 4 - Promoter induction by fetal bovine serum and forskolin. a Effect of barcode abundance on THRB-1 promoter induction by FBS or Mafb promoter induction by forskolin. P-values indicate Spearman’s rank correlation tests with Sidak adjustments for multiple comparisons. **b** Examples of spacer set effects. Sequences display the TFBMs and interspersed spacers for SRF and CREB3 promoters. Raincloud plots show SRF and CREB3 promoter responses to FBS and forskolin, respectively. **c** Examples of promoter rotation affecting promoter induction. Boxes indicate the median and interquartile range. P-values are from one-way analysis of variance tests with Sidak adjustments for multiple comparisons. **d** Fold changes for individual barcodes of the indicated THRB-1 and Mafb units following FBS and forskolin treatment, respectively. Boxes indicate the median and interquartile range. P-values are from one-way analysis of variance tests with Sidak adjustments for multiple comparisons.

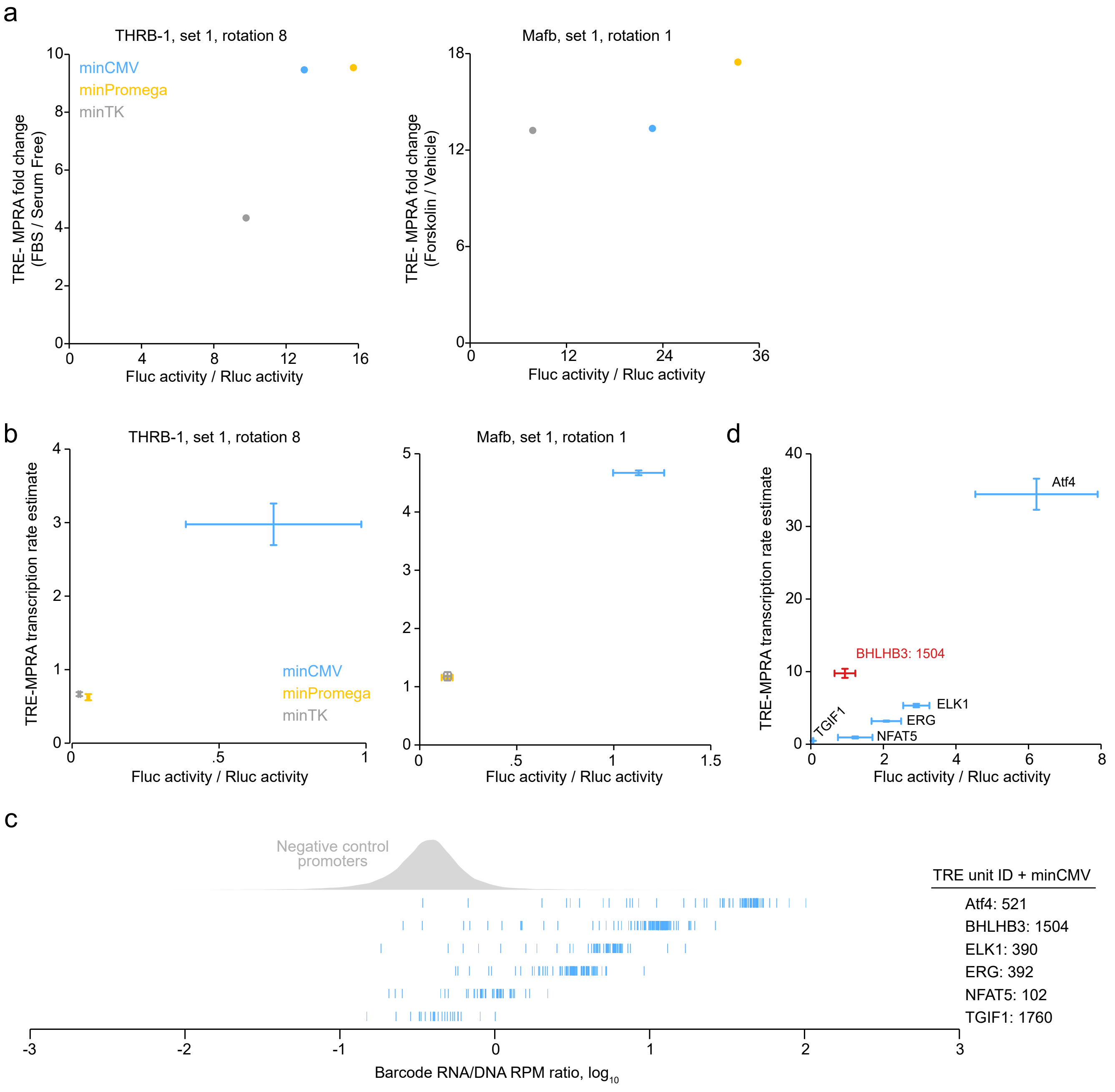

Supplementary Figure 5

Supplementary Figure 5 - Orthogonal validation of baseline transcription activities in untreated HEK293 cells. a Comparisons between the fold changes observed in the TRE-MPRA experiment and the observed raw luciferase ratios from orthogonal dual-luciferase assays for the THRB-1 and Mafb units tested in Figure 2. Luciferase ratio values are interpolated from the curves of Figure 2 at the concentrations used for the TRE-MPRA experiment. **b** Comparisons of estimated transcription rates by minimal promoter in untreated HEK293 cells from the TRE-MPRA experiment and the observed raw luciferase ratios from orthogonal dual-luciferase assays for the THRB-1 and Mafb units tested in Figure 2. Error bars indicate the standard deviation and their intersection indicates the mean. Transcription rate estimates were derived from two independent experiments. Dual-luciferase data are from three independent experimental replicates (n=3 independent experiments). **c** Raincloud plot of the average observed ratio of RNA/DNA reads per million in untreated HEK293 cells for each barcode of six promoters. The gray density plot represents the barcodes of negative controls. **d** Comparisons of estimated transcription rates from the TRE-MPRA experiment and the observed raw luciferase ratios from orthogonal dual luciferase assays for six promoters shown in B. Error bars indicate the standard deviation and their intersection indicates the mean. Transcription rate estimates were derived from two independent experiments. Dual luciferase data are from three independent experimental replicates.

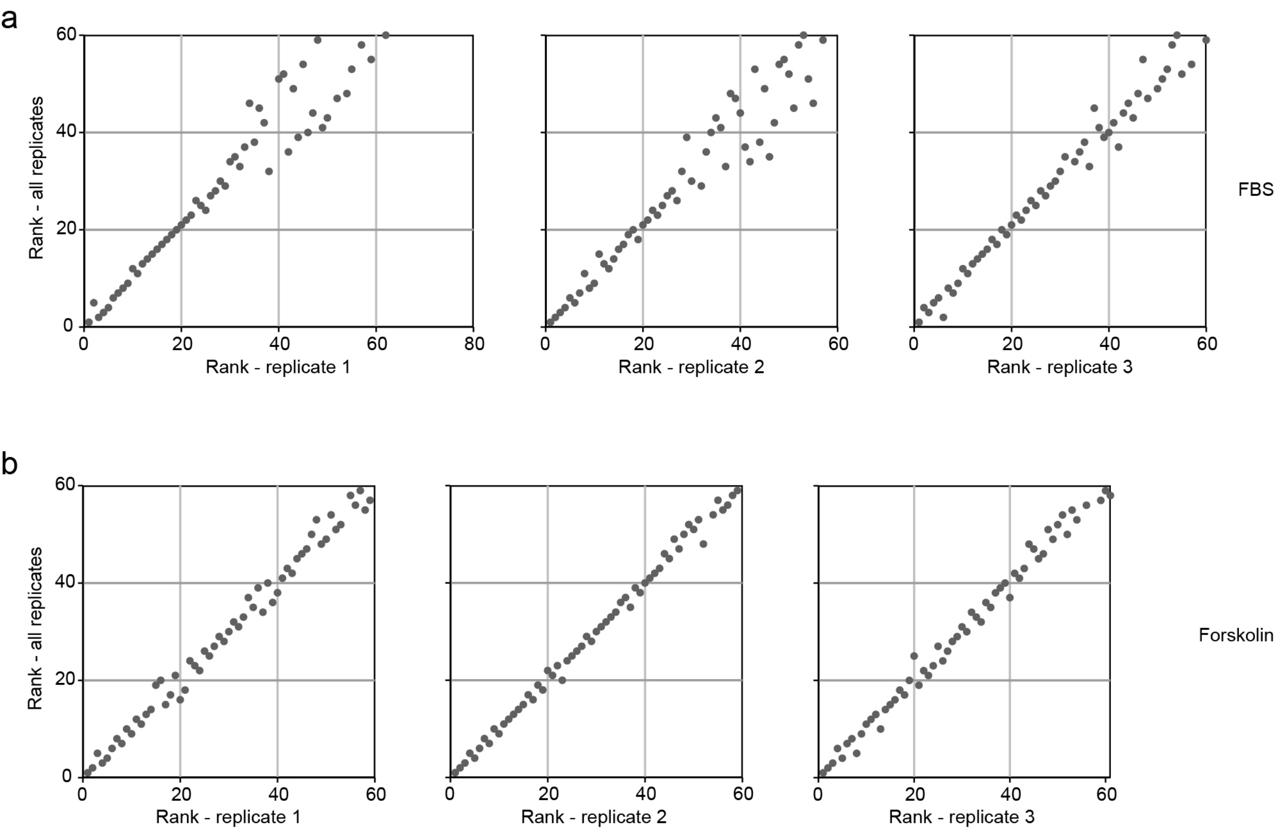

Supplementary Figure 6 - Comparing promoter rankings by single or multiple experimental replicates. a Scatterplots comparing the rankings of fold change absolute values of promoters following FBS treatment in HEK293 cells. Each individual replicate is compared to the complete group of three replicates. **b** Scatterplots comparing the rankings of fold change absolute values of promoters following forskolin treatment in HEK293 cells. Each individual replicate is compared to the complete group of three replicates.

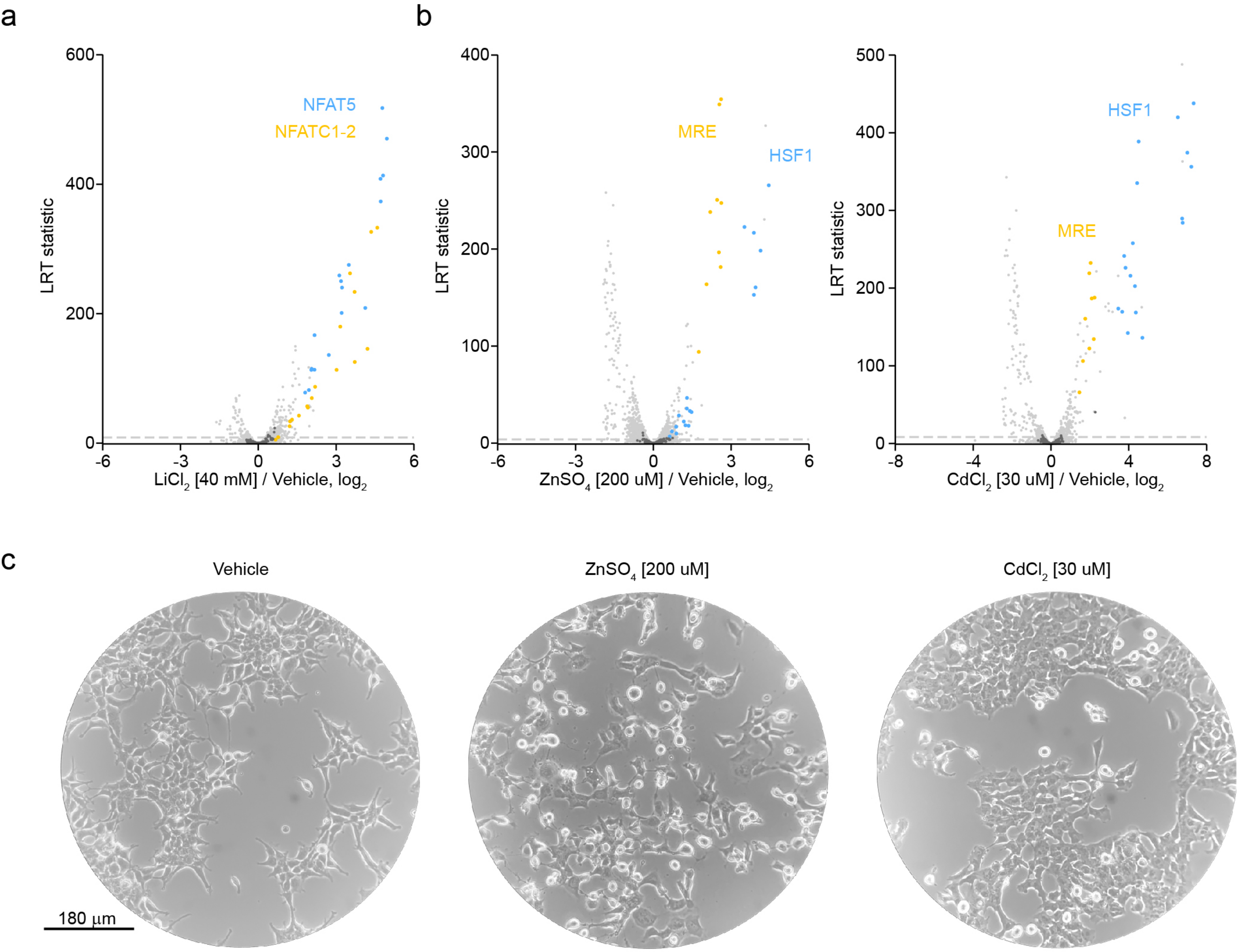

Supplementary Figure 7 - Promoter activation by exposure to lithium or heavy metals. a Volcano plots of promoter responses following treatment of HEK293 cells with 40 mM lithium chloride. **b** Volcano plots of promoter responses following treatment of HEK293 cells with 200 uM zinc sulfate or 30 uM cadmium chloride for six hours, in comparison to untreated cells. Dashed lines indicate an FDR threshold of 5%. Negative control promoters are indicated by dark gray data points. **c** Light microscope images of HEK293 cells treated with 200 uM zinc sulfate or 30 uM cadmium chloride for six hours.

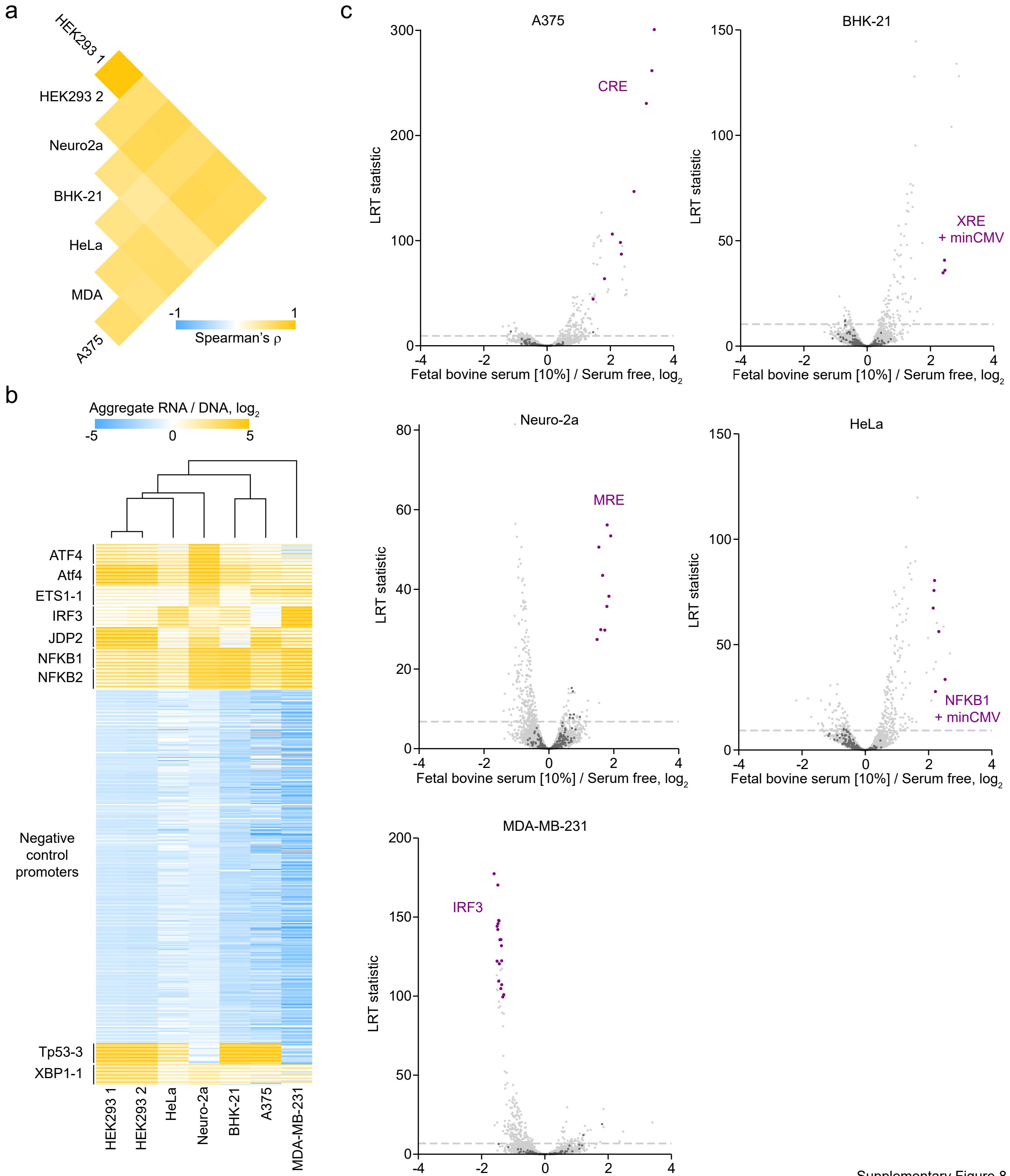

Supplementary Figure 8

Supplementary Figure 8 - Baseline promoter activity and response to fetal bovine serum in additional mammalian cell lines. a Heatmap of Spearman's rho of the estimated transcription rates for all promoters in untreated mammalian cell lines. Each pair of cell lines were positively correlated with Sidak-adjusted p-values < 0.001. **b** Heatmap of the $\log_2$ ratio of aggregate RNA and DNA barcode reads per million for selected promoters. Cell lines were clustered using Euclidean distance with complete linkage. A list of promoter (row) labels can be found in Supplementary Table 7. **c** Volcano plots of promoter responses following treatment of five mammalian cell lines with 10% FBS for six hours. Dashed lines indicate an FDR threshold of 5%. Negative control promoters are indicated by dark gray data points.

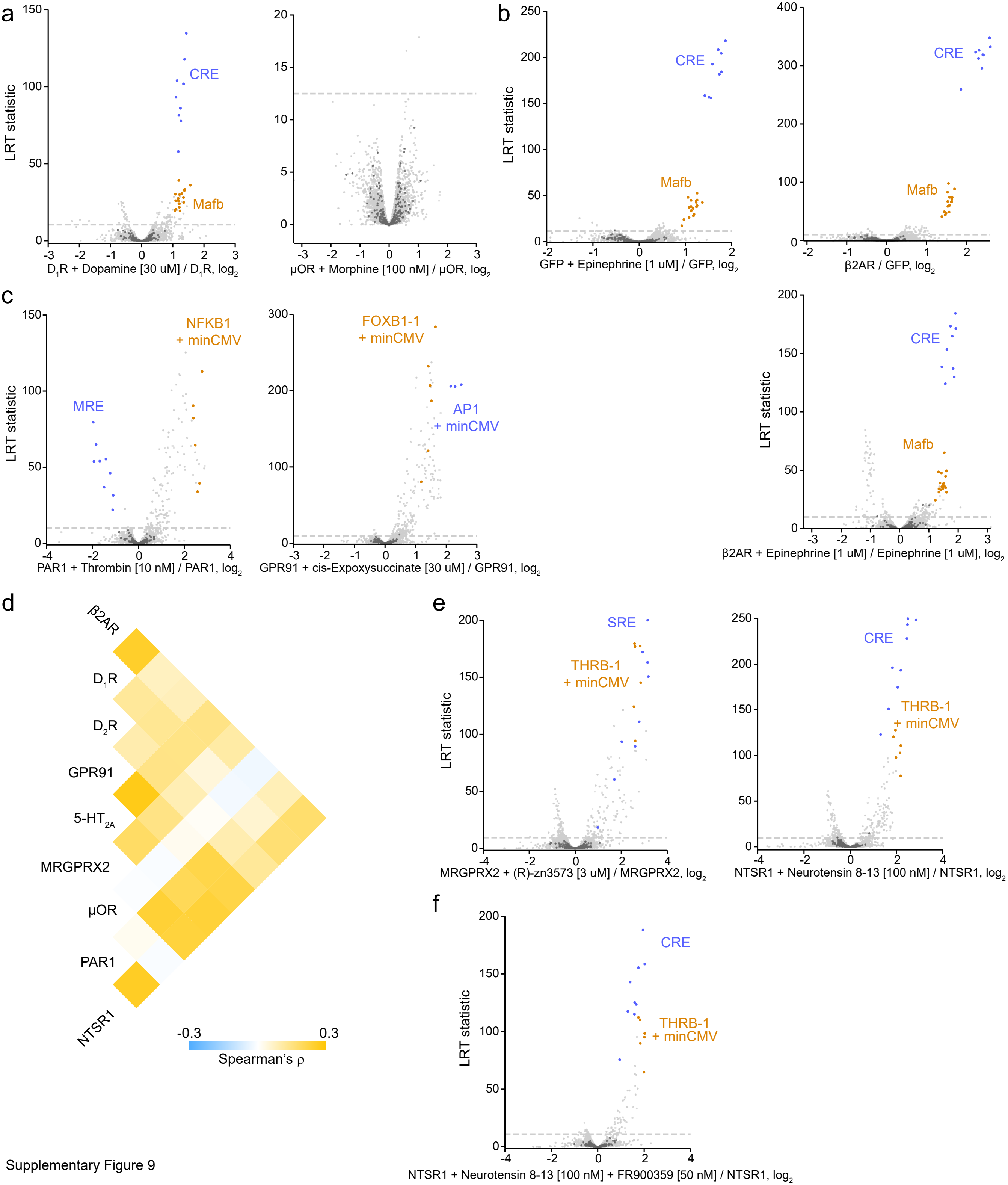

Supplementary Figure 9

Supplementary Figure 9 - Promoter responses to GPCR agonism. a Promoter responses following receptor agonism in HEK293 cells transfected with DRD1 or OPRM1 expression plasmids. Dashed lines indicate an FDR threshold of 5%. Negative control promoters are indicated by dark gray data points. **b** Promoter responses of HEK293 cells treated with 1 uM epinephrine and untreated cells and HEK293 cells transfected with GFP or ADRB2 expression plasmids. Dashed lines indicate an FDR threshold of 5%. Negative control promoters are indicated by dark gray data points. **c,e** Promoter responses in HEK293 cells transfected with GPCR expression plasmids following receptor agonism. Dashed lines indicate an FDR threshold of 5%. Negative control promoters are indicated by dark gray data points. **d** Correlations of log_2 fold change responses of GPCRs to receptor-specific agonists. **f** Promoter responses following NTSR1 agonism in FR900359 treated HEK293 cells transfected with NTSR1.

a

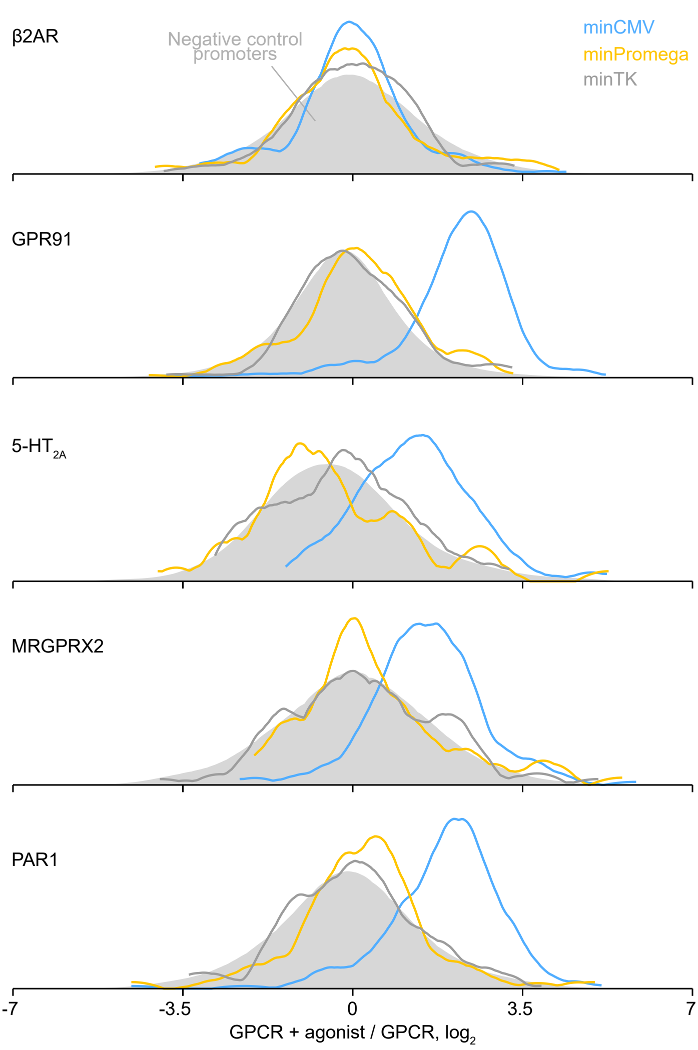
AP1 unit barcodes
β2AR
Negative control promoters
minCMV
minPromega
minTK
GPR91
5-HT2A
MRGPRX2
PAR1
-7
-3.5
0
3.5
7
GPCR + agonist / GPCR, log2

b

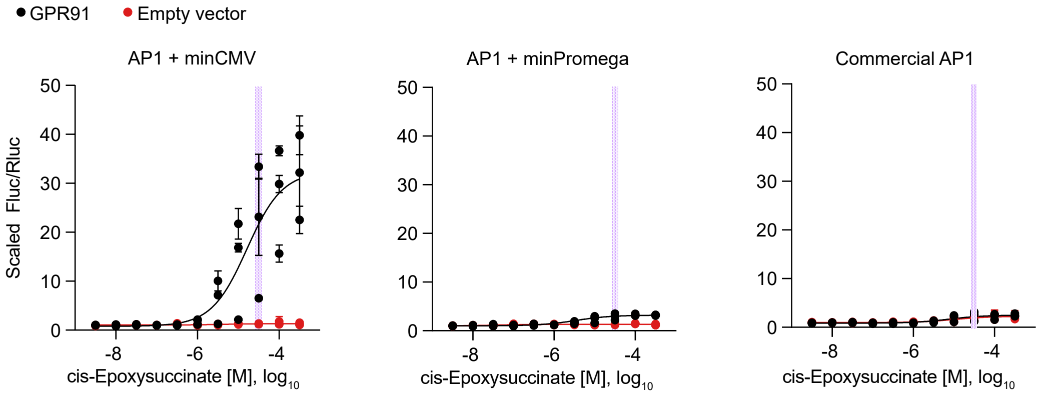
GPR91
Empty vector
AP1 + minCMV
AP1 + minPromega
Commercial AP1
Scaled Fluc/Rluc
50
40
30
20
10
0
-8
-6
-4
cis-Epoxysuccinate [M], log10

Supplementary Figure 10 – GPCR activation of AP1 promoters requires pairing with the minimal CMV promoter. **a** Density plots of AP1 unit barcode fold changes by minimal promoter following ADRB2, GPR91, 5HT2A, MRGPRX2, or PAR1 agonism. The filled gray distributions indicate the population of negative control promoter barcodes. **b** Dose response curves from HEK293 cells co-transfected with AP1 promoter (rotation of four) dual luciferase reporters and either an empty plasmid or a GPR91 expression plasmid. Data were scaled to the Fluc/Rluc ratio in untreated cells (set to a value of 1). Curves were fit to scaled Fluc/Rluc values across three experimental replicates. Data points and error bars indicate the mean and standard deviation of technical replicates within each experimental replicate (n=3 independent experiments). Shaded vertical lines indicate the drug dose used in the TRE-MPRA experiment.
