# Supplementary material for: A massively parallel reporter assay library to screen short synthetic promoters in mammalian cells
Source: Nat Commun. 2024 Nov 28;15:10353. doi: 10.1038/s41467-024-54502-9 (PMC11604768; doi:10.1038/s41467-024-54502-9)
Supplement: Supplementary file 2 — Description of Additional Supplementary Files [file 41467_2024_54502_MOESM2_ESM.pdf]

Description of Additional Supplementary Files

File Name: Supplementary Data 1
Description: Table of treatment conditions.

File Name: Supplementary Data 2
Description: Table of transcription factor binding motifs (TFBMs) used to generate candidate promoters.

File Name: Supplementary Data 3
Description: Table of TRE units.

File Name: Supplementary Data 4
Description: Table of primers.

File Name: Supplementary Data 5
Description: Table of plasmids.

File Name: Supplementary Data 6
Description: Table of promoters displayed in Figure 5B from top to bottom.

File Name: Supplementary Data 7
Description: Table of promoters displayed in Figure S8B from top to bottom.
